# Importance of Defect Detectability in Positron Emission Tomography Imaging of Abdominal Lesions

**Published:** 2015

**Authors:** Shozo Yamashita, Kunihiko Yokoyama, Masahisa Onoguchi, Haruki Yamamoto, Tetsu Nakaichi, Shiro Tsuji, Kenichi Nakajima

**Affiliations:** 1 Division of Radiology, Public Central Hospital of Matto Ishikawa, Hakusan, Japan; 2 Department of Health Sciences, Graduate School of Medical Sciences, Kanazawa University, Kanazawa, Japan; 3 PET Imaging Center, Public Central Hospital of Matto Ishikawa, Hakusan, Japan; 4 Department of Nuclear Medicine, Kanazawa University Hospital, Kanazawa, Japan

**Keywords:** Abdominal Lesion, Defect Detectability, Positron Emission Tomography

## Abstract

**Objective(s):**

This study was designed to assess defect detectability in positron emission tomography (PET) imaging of abdominal lesions.

**Methods:**

A National Electrical Manufactures Association International Electrotechnical Commission phantom was used. The simulated abdominal lesion was scanned for 10 min using dynamic list-mode acquisition method. Images, acquired with scan duration of 1-10 min, were reconstructed using VUE point HD and a 4.7 mm full-width at half-maximum (FWHM) Gaussian filter. Iteration-subset combinations of 2-16 and 2-32 were used. Visual and physical analyses were performed using the acquired images. To sequentially evaluate defect detectability in clinical settings, we examined two middle-aged male subjects. One had a liver cyst (approximately 10 mm in diameter) and the other suffered from pancreatic cancer with an inner defect region (approximately 9 mm in diameter).

**Results:**

In the phantom study, at least 6 and 3 min acquisition durations were required to visualize 10 and 13 mm defect spheres, respectively. On the other hand, spheres with diameters ≥17 mm could be detected even if the acquisition duration was only 1 min. The visual scores were significantly correlated with background (BG) variability. In clinical settings, the liver cyst could be slightly visualized with an acquisition duration of 6 min, although image quality was suboptimal. For pancreatic cancer, the acquisition duration of 3 min was insufficient to clearly describe the defect region.

**Conclusion:**

The improvement of BG variability is the most important factor for enhancing lesion detection. Our clinical scan duration (3 min/bed) may not be suitable for the detection of small lesions or accurate tumor delineation since an acquisition duration of at least 6 min is required to visualize 10 mm lesions, regardless of reconstruction parameters. Improvements in defect detectability are important for radiation treatment planning and accurate PET-based diagnosis.

## Introduction

Positron emission tomography (PET) with ^18^F-fluorodeoxyglucose (^18^F-FDG) is a useful imaging method for distinguishing benign abdominal tumors from malignant ones. For this purpose, detection of hot lesions is of high significance for quantitative and qualitative analyses of tumors.

Several studies have shown that the detection of hot lesions is influenced by various factors such as the PET scanner, reconstruction parameters, scanning parameters, and body habitus (1-7). Japanese guidelines for oncology FDG-PET/computed tomography (CT) have specified certain criteria for detecting hot lesions in order to standardize PET image quality in various PET centers and different PET camera models (8). With regard to radiation therapy planning, adequate detection is necessary for the accurate delineation of target volumes (9-11).

On the other hand, lesions are described as defects when FDG uptake is lower than that of the surrounding tissues. In addition, detection of these lesions is helpful for tumor diagnosis since most benign abdominal lesions show poor uptake (12-16). However, lesions, particularly small ones, may not be detected given the overlapping radioactivity from the background (BG).

Moreover, respiratory motion results in image blurring and inaccurate attenuation correction, caused by misregistration between PET and CT data, which leads to misdiagnosis. There have been only a few reports on defect detection of tumors, despite the clinical importance. Therefore, the aim of this study was to assess defect detectability in positron emission tomography (PET) imaging of abdominal lesions, using phantom and clinical studies.

## Methods

### 

#### Phantom study

A National Electrical Manufactures Association 2001 International Electrotechnical Commission phantom was used (Data Spectrum Corp., Hillsborough, NC). This phantom consisted of a torso cavity, a removable lung insert, and six spheres with the inner diameters of 10, 13, 17, 22, 28, and 37 mm. The spheres were filled with non-radioactive water, and BG was set to 2.65 kBq/mL, which is similar to clinical abdominal conditions.

#### Data acquisition and image reconstruction

PET/CT scans were performed using Discovery PET/CT 600 Motion Scanner (GE Healthcare, Milwaukee, WI), and a 3-dimensional (3D)-only scanner. The phantom was scanned for 10 min, using dynamic list-mode acquisition method. Images acquired using scan duration of 1-10 min were reconstructed, using a 3D ordered subset expectation maximization (OSEM) algorithm with VUE point HD and a 4.7-mm full-width at half-maximum (FWHM) Gaussian filter.

The iteration-subset combinations of 2-16 and 2-32 were used. The transaxial field of view (FOV) was 550 mm, the slice thickness was 3.27 mm, and the matrix size was 128×128. Attenuation correction was performed, using a 16-slice CT scanner. The scanning parameters were as follows: 120 kVp, 10-80 mA, noise index 20, rotation time 0.6 s, pitch 1.75:1, slice thickness 3.75 mm, transaxial FOV 700 mm, and matrix size 512×512.

#### Data analysis

Advantage Workstation Version 4.4 (GE Healthcare, Milwaukee, WI) was used for visual and physical analyses. For visual analysis, the defect areas were evaluated by two experts including a certified PET physician at the Japanese Society of Nuclear Medicine and a certified PET technologist at the Japanese Society of Nuclear Medicine Technology.

We used the center slice where the spheres were most prominent. The images were displayed using an inverse grayscale with the standardized uptake range of 0-4. These defect spheres were visually graded as follows: identifiable (2), scarcely identifiable (1), and unreadable (0). A sphere could be visualized when the average score was reported to be ≥1 by the two experts.

For physical indices, mean radioactivity *C_D,j_* (kBq/mL) of six defect spheres *j* was determined using a region of interest (ROI) of the same diameter. BG was determined using 12 ROIs of the same diameter with six defect spheres in the center slice, and the average of mean radioactivity *C_B12,j_* (kBq/mL) was calculated. The defect contrast was calculated, using the following formula:


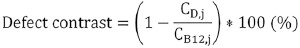


In addition, 12 ROIs were set up in four additional slices (±1 and ±2 cm of the upper and lower sides of the center slice, respectively), and the percentage of BG variability was calculated using a total of five slices and the average value of 60 ROIs (*C_B60,j_*):


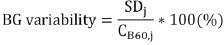


Standard deviation (SD) was calculated as follows:


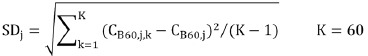


Considering the statistical variation of PET images, defect contrast and BG variability were calculated based on the average of three images, which were reconstructed 0, 1, and 2 min after the point of initiation.

#### Clinical study

To sequentially evaluate defect detectability in clinical settings, we examined two male subjects (60 and 50 years old, respectively). One of them had a liver cyst (9.4×10.4 mm in diameter) in the right lobe and the other suffered from pancreatic cancer with an inner defect region in the pancreatic body, suggesting necrosis. The inner defect size was 9.1×8.7 mm in diameter, measured by enhanced CT, performed one week before PET/CT examination.

After the subjects fasted for at least 5 hours, FDG was intravenously injected with 4.2 and 4.1 MBq/kg radioactivity, respectively. The PET scans were performed at 138 and 140 min after the injection, respectively. The lesions were scanned for 10 min using the dynamic list-mode acquisition method. During the PET and CT scans, the patients were freely breathing. CT scan parameters were as follows: 120 kVp, 10-200 mA, noise index 10, rotation time 0.6 s, pitch 1.75:1, slice thickness 3.75 mm, transaxial FOV 500 mm, and matrix size 512×512.

This study was approved by the ethics committee of our institution. Written informed consents were obtained from all patients.

#### Statistical analysis

Regression analysis and Pearson’s correlation coefficient were used to assess the association between visual scores and physical indices. *P*-value < 0.05 was considered statistically significant.

## Results

### 

#### Phantom study

**Figure 1:**
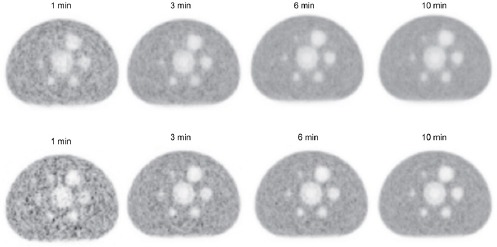
Phantom images acquired using scan duration 1, 3, 6 and 10 min. *Upper row:* positron emission tomography (PET) images were reconstructed using the iteration–subset combination 2–16. *Lower row:* PET images were reconstructed using the iteration–subset combination 2–32. All images were reconstructed using a 4.7-mm full-width at half-maximum Gaussian filter

**Figure 2:**
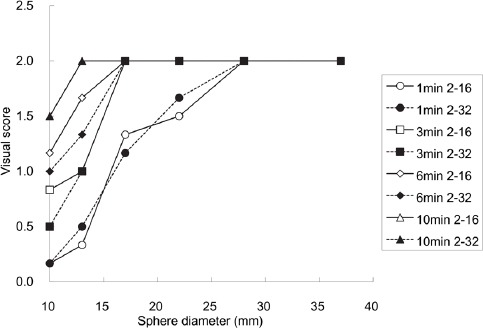
The relationship between sphere diameter and the visual score depending on acquisition duration. The visual scores of images acquired for 10 min were identical between iteration–subset combinations 2–16 and 2–32

The scanned images are shown in Figure 1. The relationship between sphere diameter and visual score, depending on acquisition duration, is indicated in Figure 2. For the 10 mm defect sphere, acquisition duration of ≥6 min was required to achieve a visual score of ≥1, regardless of iteration-subset combinations. For the 13 mm defect sphere, acquisition duration of ≥3 min was necessary to achieve a visual score of ≥1. The visual scores of spheres ≥17 mm were >1, even if the acquisition duration was only 1 min.

**Figure 3:**
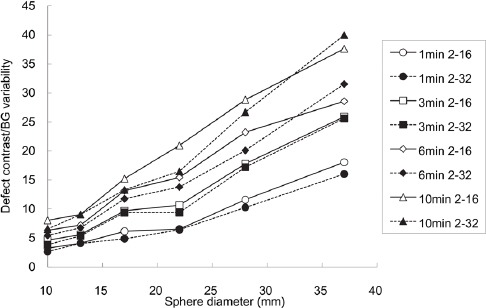
The relationship between sphere diameter and defect contrast/background (BG) variability depending on acquisition duration

The relationship between sphere diameter and defect contrast/BG variability, depending on acquisition duration, is demonstrated in Figure 3. The defect contrast/BG variability increased as the sphere diameter and acquisition duration increased. The sets of curves for the two reconstruction parameters were almost identical. Regarding the visible spheres in the visual evaluation, all defect contrast/BG variability values were approximately ≥ 5.

**Figure 4:**
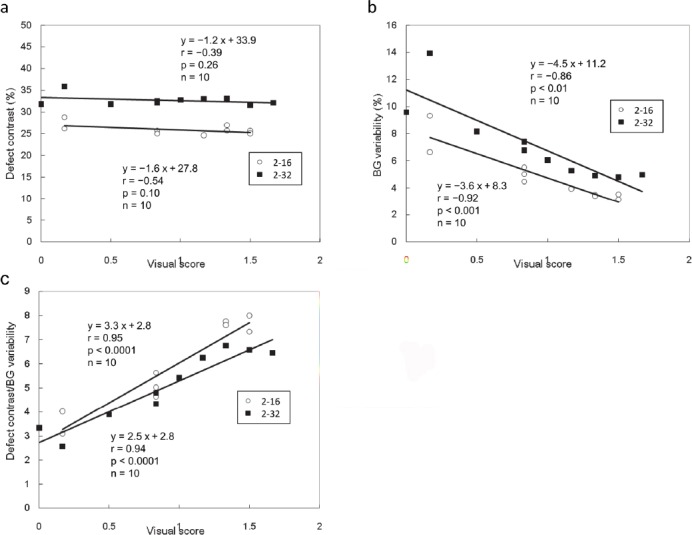
The relationship between visual score and physical indices such as defect contrast (a), BG variability (b), and defect contrast/BG variability (c) for the 10-mm sphere. ○ the iteration–subset combination 2–16, ◼the iteration-subset combination 2–32

For the 10 mm defect sphere, the relationship between visual score and physical indices is shown in Figure 4. As the results indicated, the visual score was not significantly correlated with defect contrast (2-16: *r*=−0.54, *P*=0.10; 2-32: *r*=−0.39, *P*=0.26). Contrarily, the visual score was significantly correlated with BG variability (2-16: *r*=−0.92, *P*<0.001; 2-32: *r*=−0.86, *P*<0.01) and defect contrast/BG variability (2-16: *r*=0.95, *P*<0.0001; 2-32: *r* = 0.94, *P*<0.0001).

#### Clinical study

**Figure 5:**
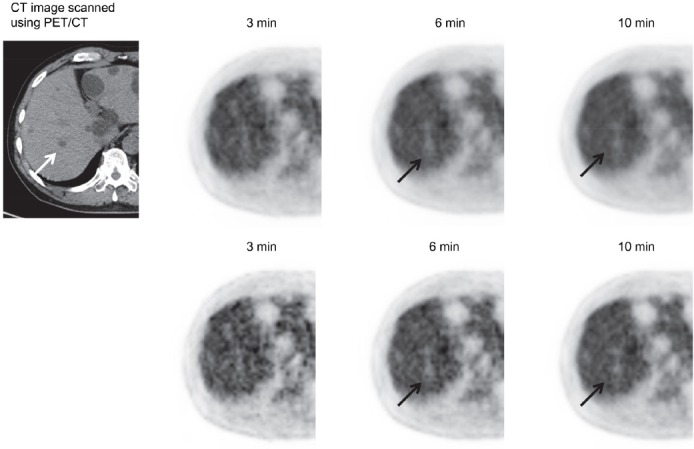
A 60-year-old male patient with a liver cyst (approximately 10 mm in diameter) in the right lobe (*arrow*). PET images acquired at scan durations of 3, 6, and 10 min. *Upper row:* Images reconstructed using the iteration–subset combination 2–16; *lower row:* Images reconstructed using the iteration–subset combination 2–32. Although the defect corresponding to the cyst was not detected at the acquisition duration of 3 min; this defect was slightly observed at 6 min, regardless of reconstruction parameters

**Figure 6:**
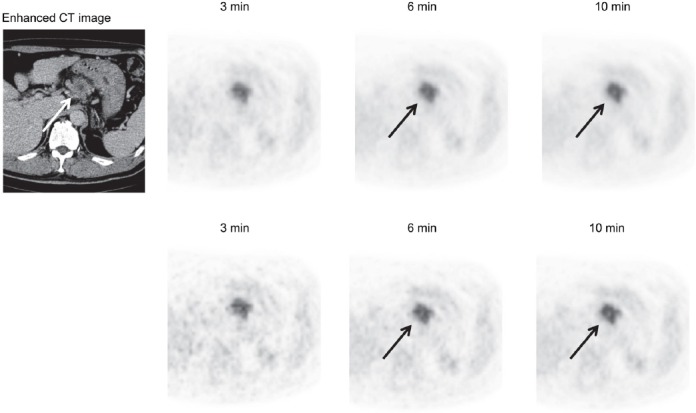
A 50-year-old male patient with pancreatic cancer that contained an inner defect region (approximately 9 mm in diameter) (*arrow*). PET images acquired using scan durations of 3, 6, and 10 min. *Upper row:* Images reconstructed using the iteration–subset combination 2–16; *lower row:* Images reconstructed using the iteration–subset combination 2–32. The unenhanced inner small region, suggesting necrosis, was clearly observed as the acquisition duration increased

Figure 5 shows a patient with a small liver cyst. Although the defect of the cyst was not observed at the acquisition duration of 3 min, it was slightly visualized at 6 min. Figure 6 shows a patient with pancreatic cancer and an inner defect region. Although the small defect region was not observed at the acquisition duration of 3 min, it was clearly detected when the acquisition duration was increased.

## Discussion

Detection of myocardial defects has been evaluated in several studies (17-19). Matsunari et al. (19) showed a significant correlation between the measured and true defect size using a chest phantom; also, PET could accurately quantify the myocardial defect size. Therefore, PET is useful for detecting not only hot lesions but also defect lesions.

Recently, PET has been applied to evaluate intraductal papillary mucinous neoplasms of the pancreas and mucinous cystic neoplasms to decide whether to perform resection (14) (20-22). Most of these lesions are small in size and may not be detected as defects even if there is no uptake. Problems associated with defect detection include overlapping radioactivity from BG, image blurring, and inaccurate attenuation correction, caused by respiratory motion. However, there are limited reports on the defect detectability in tumors. Therefore, this study was designed to assess defect detectability in positron emission tomography (PET) imaging of abdominal lesions.

Improvements in defect detectability are important for distinguishing the uptake of a tumor itself from that of artifacts when the lesion uptake is equal to BG radioactivity. It is also useful when the visual analysis of tumor activity is required; for instance, we could determine whether the tumor is a cyst or some other type of lesion such as a well-differentiated hepatocellular carcinoma. Furthermore, for a cystic tumor, uptake detection in the rim and mural nodule is necessary for qualitative diagnosis (22, 23), and this type of uptake could be easily identified by improving defect detectability.

The phantom study results showed that acquisition durations of at least 6 and 3 min are required for visualizing 10 and 13 mm defect spheres, respectively. In clinical settings, a cyst of approximately 10 mm in diameter can be visualized with the acquisition duration of 6 min, although the image has suboptimal quality. According to these findings, 10 mm defect lesions may not be detected at the emission duration of 3 min/bed, which is the typical duration in clinical scanning. However, lengthy acquisition is not desirable for clinical use since it may be a burden on patients and delay the study schedule.

Some studies have shown that techniques such as time-of-flight and point-spread function improve defect detectability (24) and may resolve the associated problems. On the other hand, the present results showed that a 17-mm sphere can be detected even if the acquisition duration is only 1 min. Consequently, if the uptake is observed in a lesion with a diameter of ≥17 mm, it is considered a true tumor uptake.

Our study was conducted using two reconstruction parameters including iteration–subset combinations of 2–16 and 2–32. Although the defect contrast of the combination 2–32 is higher than that of 2–16, the BG variability of 2–32 is inferior to that of 2–16. Accordingly, the two reconstruction parameters were compared in terms of defect contrast/BG variability, which was used as an indicator similar to signal-to-noise ratio.

The results showed that the curves of two reconstruction parameters are nearly identical when the acquisition duration is similar (Figure 3). The visual scores also showed a similar tendency. Therefore, these reconstruction parameters are assumed to yield almost the same detectability of defect lesions. In addition, all the visible spheres show a defect contrast/BG variability of approximately ≥5. This value could be an indicator of adequate defect detectability, although its universal application should be studied in different conditions.

With respect to the relationship between visual score and physical indices, the visual score was significantly correlated with BG variability (Figure 4b). These results showed that the reduction of BG variability is the most important factor for enhancing the detection of defect lesions.

During radiation therapy planning, defect detection is useful for accurate tumor delineation. According to the present clinical study, acquisition duration of 3 min is insufficient for the detection of small inner defect regions. This finding indicates that our clinical scan duration may not be optimal for the correct delineation of a small defect region in a tumor. Longer acquisition duration (6-10 min) is also useful for enhancing detectability.

This study had several limitations. Our phantom study was performed using BG radioactivity that is similar to our clinical abdominal conditions in a delayed scan. However, defect detectability may be affected by BG radioactivity, as reported by Brambilla and colleagues (25). Moreover, lesions including slight uptake were not considered although the uptake was lower than that of the surrounding tissues. Therefore, further phantom studies utilizing different levels of BG radioactivity and varying ratios of defect and BG radioactivity are required.

In addition, our phantom study was performed using a motionless phantom. The image quality in clinical settings is inferior to that of a phantom study due to misregistration between PET and CT data, caused by respiratory motions. The respiratory-gated, deep-inspiration, breath-hold acquisition methods may be good options for reducing these undesirable effects (26, 27). In clinical settings, further studies on more patients with various types of abdominal lesions are required.

## Conclusion

The improvement of BG variability is the most important factor for enhancing the detection of defect lesions. Our clinical scan duration (3 min/bed) may not be optimal for the detection of small defect lesions or accurate tumor delineation since an acquisition duration of at least 6 min is required to visualize 10 mm defect lesions, regardless of reconstruction parameters. Improvement of defect detectability is important for accurate PET diagnosis and radiation therapy planning.
